# Water–Fat Separated T1 Mapping in the Liver and Correlation to Hepatic Fat Fraction

**DOI:** 10.3390/diagnostics13020201

**Published:** 2023-01-05

**Authors:** Claudia Fellner, Marcel Dominik Nickel, Stephan Kannengiesser, Niklas Verloh, Christian Stroszczynski, Michael Haimerl, Lukas Luerken

**Affiliations:** 1 Department of Radiology, University Hospital Regensburg, 93053 Regensburg, Germany; 2 MR Application Predevelopment, Siemens Healthcare, 91052 Erlangen, Germany; 3 Department of Diagnostic and Interventional Radiology, Medical Center University of Freiburg, 79106 Freiburg, Germany

**Keywords:** liver, quantitative magnetic resonance imaging, T1 mapping, Dixon

## Abstract

(1) Background: T1 mapping in magnetic resonance imaging (MRI) of the liver has been proposed to estimate liver function or to detect the stage of liver disease, among others. Thus far, the impact of intrahepatic fat on T1 quantification has only been sparsely discussed. Therefore, the aim of this study was to evaluate the potential of water–fat separated T1 mapping of the liver. (2) Methods: A total of 386 patients underwent MRI of the liver at 3 T. In addition to routine imaging techniques, a 3D variable flip angle (VFA) gradient echo technique combined with a two-point Dixon method was acquired to calculate T1 maps from an in-phase (T1_in) and water-only (T1_W) signal. The results were correlated with proton density fat fraction using multi-echo 3D gradient echo imaging (PDFF) and multi-echo single voxel spectroscopy (PDFF_MRS). Using T1_in and T1_W, a novel parameter FF_T1 was defined and compared with PDFF and PDFF_MRS. Furthermore, the value of retrospectively calculated T1_W (T1_W_calc) based on T1_in and PDFF was assessed. Wilcoxon test, Pearson correlation coefficient and Bland–Altman analysis were applied as statistical tools. (3) Results: T1_in was significantly shorter than T1_W and the difference of both T1 values was correlated with PDFF (R = 0.890). FF_T1 was significantly correlated with PDFF (R = 0.930) and PDFF_MRS (R = 0.922) and yielded only minor bias compared to both established PDFF methods (0.78 and 0.21). T1_W and T1_W_calc were also significantly correlated (R = 0.986). (4) Conclusion: T1_W acquired with a water–fat separated VFA technique allows to minimize the influence of fat on liver T1. Alternatively, T1_W can be estimated retrospectively from T1_in and PDFF, if a Dixon technique is not available for T1 mapping.

## 1. Introduction

In most clinical applications, magnetic resonance imaging (MRI) is used as a qualitative imaging modality. Quantitative assessment of T1 relaxation times is increasingly discussed as a valuable tool for the detection and staging of liver disease. Compared with T1-weighted images, whose signal intensities are—among others—dependent on the T1 relaxation times of different tissues, T1 maps are independent of other contrast mechanisms, scanner hardware or software [1]. T1 mapping thus enables quantitative inter-individual comparisons as well as intra-individual follow-up examinations.

Unenhanced T1 mapping of the liver was proposed by some authors to discriminate patients with liver cirrhosis from those with normal liver function [2,3,4,5], a correlation of native T1 with albumin was found even in non-cirrhotic liver parenchyma, suggesting a direct influence of liver’s synthesis capacity on T1 relaxation times [6]. Most work, however, has been performed using a liver-specific contrast agent (Gd-EOB-DTPA, gadoxetic acid). The accumulation of the paramagnetic contrast agent in the liver parenchyma increases the signal intensity on T1-weighted images with a maximum in the hepatobiliary phase, 20 min after injection [7]. Several authors have reported a close correlation between Gd-EOB-DTPA-enhanced T1 (or the reduction rate of T1) and the results of the indocyanine green clearance test, a well-established method for the evaluation of liver function [8,9,10,11]. Kamimura et al. [8] and Haimerl et al. [11] were able to show even superior results of T1 relaxometry compared to results from dedicated signal intensity-based indices. Furthermore, a possible role of Gd-EOB-DTPA-enhanced T1 mapping for the detection and classification of liver fibrosis has been discussed in comparison to clinical scores such as the Child–Pugh score [5,8,12,13] or the Model for End-stage Liver Disease score [12,14] as well as to the histology-based METAVIR score for liver fibrosis and cirrhosis [15,16]. Recently, differentiation of liver lesions using Gd-EOB-DTPA [17] or Gd-DTPA [18] and detection of hepatocellular carcinoma (HCC) recurrence after hepatectomy [19] or even HCC grading [20] have been described using the T1 reduction rate.

Hepatic steatosis and steatohepatitis are common histologic manifestations of several liver diseases [21]. Nevertheless, the influence of fat on T1 relaxometry in the liver has only been sparsely discussed so far. Depending on the acquisition technique and sequence parameters used for T1 mapping, the presence of intrahepatic fat can result in a reduction or even prolongation of measured T1 [22,23]. Based on phantom measurements and a series of 15 patients, Le et al. concluded that Dixon water-only images yield more reliable results for T1 in the presence of fat [22]. Water-only T1 mapping was also reported to be superior for the evaluation of liver capacity in a single study [24].

Therefore, the purpose of our work was to apply water–fat separated T1 quantification in the liver using a variable flip angle (VFA) technique [25] in combination with a Dixon technique [26] in a large patient collective and to compare T1 calculated from an in-phase (T1_in) and water-only (T1_W) signal depending on the intrahepatic fat fraction. Furthermore, we aimed to show if T1_W can be estimated retrospectively to minimize the influence of fat on liver T1 using T1_in and proton density fat fraction (PDFF).

## 2. Materials and Methods

### 2.1. Patients

This is a retrospective secondary analysis of data acquired in an observational study at the Department of Radiology of the University Hospital Regensburg; the study was approved by the local institutional review board; all patients gave informed consent.

In total, 406 successive magnetic resonance (MR) examinations of the liver which were conducted according to our study protocol were analyzed retrospectively. MR of the liver was carried out for the assessment of suspected liver lesions or as a follow-up in case of known liver disease.

### 2.2. MR Data Acquisition

MR was performed on a clinical 3 T scanner (MAGNETOM Skyra, Siemens Healthcare, Erlangen, Germany) using an 18-channel body coil and elements of a 32-channel spine coil for signal reception. Standard liver imaging included T2-weighted Turbo Spin Echo (TSE) sequences with and without spectral fat saturation (fs) as well as diffusion-weighted imaging; a 3D Volumetric Interpolated Breathhold Examination (VIBE) fs sequence was acquired precontrast as well as in the arterial, portal venous, late venous and hepatobiliary phase 20 min after injection of Gd-EOB-DTPA (Primovist, Bayer Healthcare, Berlin, Germany; dosage: 0.1 mL/kg bodyweight). Mapping of T1, fat fraction and R2* of the whole liver as well as multi-echo single voxel spectroscopy were added in all patients.

T1 mapping was performed with a VFA prototype 3D VIBE sequence during breathhold: Repetition time (TR) = 5.79 ms, echo times (TE) = 2.46, 3.69 ms, flip angles = 1, 7, and 14°, measured voxel size 3.6 mm × 2.5 mm × 4.8 mm (interpolated to 1.3 mm × 1.3 mm × 3.0 mm), with an acceleration factor of 4 using Controlled Aliasing In Parallel Imaging Results IN Higher Acceleration (CAIPIRINHA) [27] and acquisition time of 17 s. A preceding B1 map was measured for inline correction of B1 inhomogeneities. T1 maps from in-phase (T1_in) and water (T1_W) signal were calculated inline after fat–water separation by a 2-point Dixon method.

Simultaneous mapping of PDFF and R2* was carried out with a 6-echo prototype 3D VIBE sequence using a multi-step adaptive fitting approach which accounts for the spectral complexity of fat [28]: TR = 9.2 ms, TE = 1.23 to 7.38 ms with ΔTE = 1.23 ms, flip angle = 4°, measured voxel size 2.9 mm × 2.6 mm × 6.4 mm (interpolated to 2.6 mm × 2.6 mm × 4.0 mm), CAIPIRINHA with acceleration factor of 4 and acquisition time of 15 s. A region of interest (ROI) was positioned manually on morphological images in an area free of liver lesions and major vessels; this ROI was transferred automatically to PDFF and R2* maps for inline evaluation of PDFF and R2*.

Single voxel breath-hold ^1^H MRS (SVS) for fat quantification was acquired using a Stimulated Echo Acquisition Mode (STEAM) sequence with 5 TEs in 5 concatenated TRs: TR = 3000 ms, TE = 12, 24, 36, 48, and 72 ms, voxel size 30 mm × 30 mm × 30 mm, sampling points = 1024, acquisition time = 15 s. The voxel was co-localized with the ROI for PDFF quantification. Inline reconstruction and assessment resulted in a spectrum with T2 correction and the corresponding fat fraction (PDFF_MRS) [29].

### 2.3. MR Data Analysis

MR examinations with global fat–water swaps in T1 and/or fat mapping were excluded from further evaluation. As severe motion artifacts were most clearly seen on PDFF maps as bright and dark ripples, we also excluded patients if PDFF was above 2% and the standard deviation of PDFF in the ROI was larger than 100%. Furthermore, we excluded patients with R2* of the liver above 200 s^−1^ because SVS included quite long echo times which will result in very low signal and confounded PDFF values in case of considerably increased liver iron content.

An ROI corresponding to the ROI for PDFF and R2* analysis was drawn manually on T1_in and T1_W maps. Using T1_in and T1_W, a novel parameter FF_T1 was defined as

FF_T1 = (T1_W-T1_in)/(T1_in) 100(1)

FF_T1 was correlated with PDFF and PDFF_MRS to assess its value as a measure of the fat fraction.

### 2.4. Statistical Analysis

Statistical analyses were performed using SPSS 25.0 (IBM SPSS Statistics, Armonk, New York, NY, USA) and GraphPad Prism 7.0 (GraphPad Software Inc., San Diego, CA, USA). Means and standard deviations were calculated for all data, and the Wilcoxon test was applied to evaluate differences between T1_in and T1_W. Pearson’s correlation coefficient R was calculated for the parameters T1_W-T1_in and PDFF, for PDFF, PDFF_MRS, and FF_T1 as well as for T1_W and T1_W_calc. Furthermore, Bland–Altman analysis [30] was performed to compare PDFF, PDFF_MRS, and FF_T1 as well as T1_W and T1_W_calc. For all statistical tests, results with *p* < 0.05 were considered to be statistically significant.

## 3. Results

A total of 427 MR examinations were analyzed retrospectively: 21 of them had to be excluded due to global fat–water swaps in T1 mapping (n = 7), PDFF mapping (n = 3) or swaps in both methods (n = 11). Sixteen examinations were excluded because of severe motion artifacts and four were excluded because R2* was above 200 s^−1^: R2* was 207.6, 244.4, 308.7 and 553.3 s^−1^ in those patients. From the remaining 386 examinations, 264 were performed in men, and 122 were performed in women; the mean age was 58 ± 18 years (age range: 18 to 85 years).

Overall, T1_W was significantly longer (*p* < 0.001) compared to T1_in. In two patients, T1_W and T1_in were identical; in another eight patients, T1_in was longer than T1_W with a maximum difference of 7 ms (Table 1).

**Figure 1:**
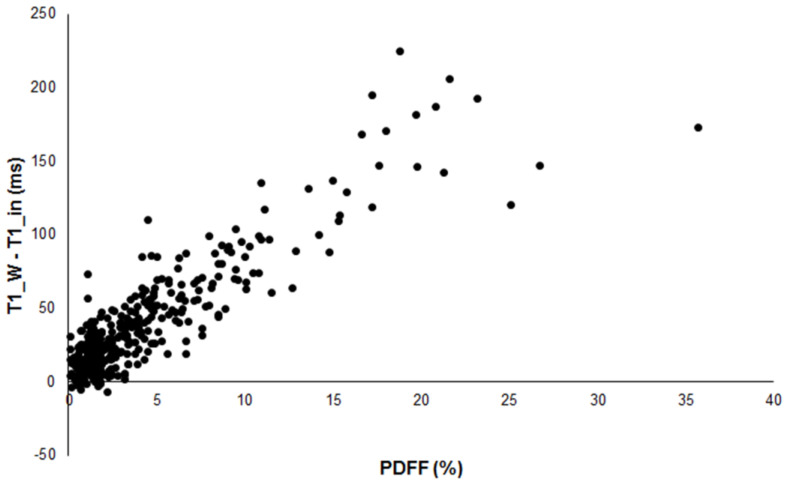
Scatterplot of the difference between T1_W and T1_in versus PDFF in 386 patients showing good correlation (R = 0.890).

In those 10 patients, PDFF and PDFF_MRS were between 0 and 2.5%. The difference T1_W-T1_in showed a significant correlation (*p* < 0.001) with PDFF with a correlation coefficient of 0.890 (Figure 1).

The majority of our patients revealed rather low PDFF and R2* within the liver: PDFF was below 5% in 292 patients and between 5 and 10% in 58 patients; PDFF between 10 and 20% was found in 29 patients and 7 patients showed PDFF above 20%. R2* was below 100 s^−1^ in 372 patients and above 100 s^−1^ in 14 patients. Details for PDFF, PDFF_MRS, FF_T1 and R2* are summarized in Table 1.

**Figure 2:**
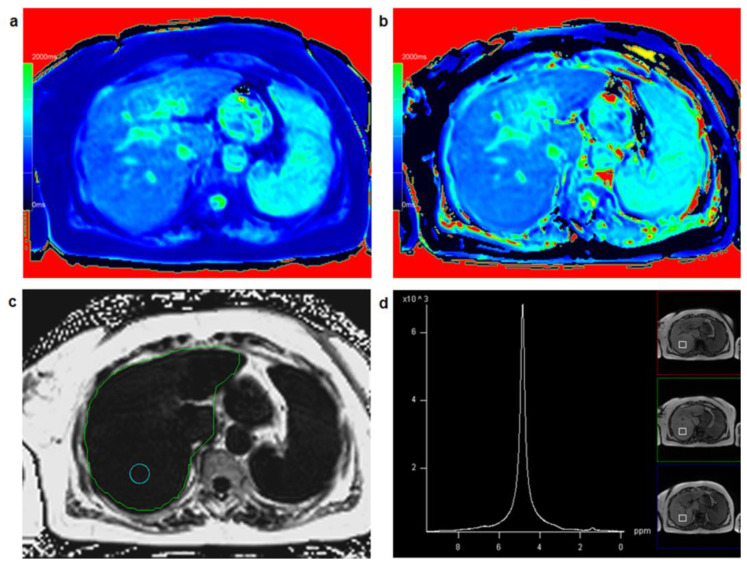
A 67-year-old female patient: T1 mapping from in-phase signal (**a**) and water-only signal (**b**) yielded similar T1 relaxation times for T1_in (=912 ms) and T1_W (=929 ms) suggesting low fat fraction in the liver with a fat fraction calculated from T1 mapping FF_T1 = 1.9%. Comparing T1_in and T1_W maps with identical window and center settings yields very similar color impression of the liver. PDFF mapping in a circular region of interest (**c**) resulted in PDFF = 1.1%, PDFF_MRS in a co-localized voxel (**d**) was 1.7%.

**Figure 3:**
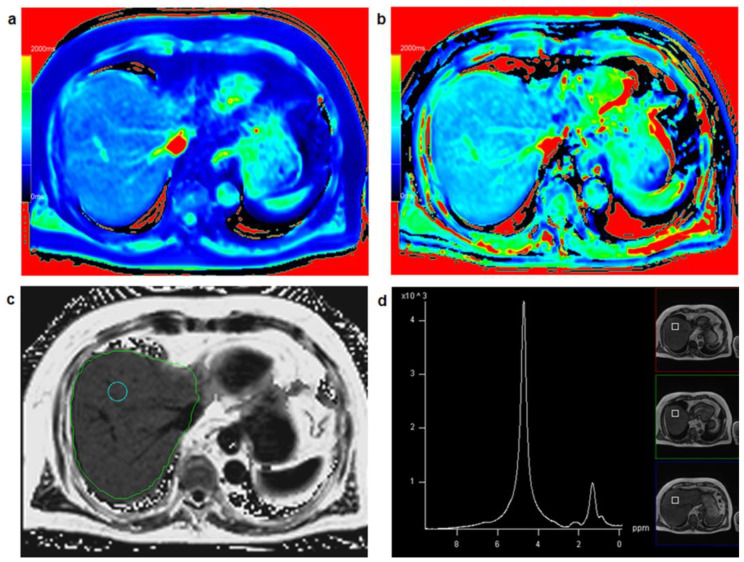
**A** 75-year-old male patient: T1 mapping from in-phase signal (**a**) and water-only signal (**b**) yielded clearly different T1 relaxation times for T1_in (=733 ms) and T1_W (=880 ms) suggesting increased fat fraction in the liver with a fat fraction calculated from T1 mapping FF_T1 = 20.1%. Comparing T1_in and T1_W maps with identical window and center setting yields clearly different color impression of the liver. PDFF mapping (**c**) resulted in PDFF = 17.6%, PDFF_MRS in a co-localized voxel (**d**) was 19.6%.

Figure 2 shows a patient with low hepatic fat fraction and very similar results for T1_in and T1_W while Figure 3 depicts a patient with increased PDFF and clearly different results between T1_in and T1_W.

PDFF, PDFF_MRS and FF_T1 showed a very high correlation coefficient (Table 2), all correlations were significant at the *p* < 0.001 level.

**Figure 4:**
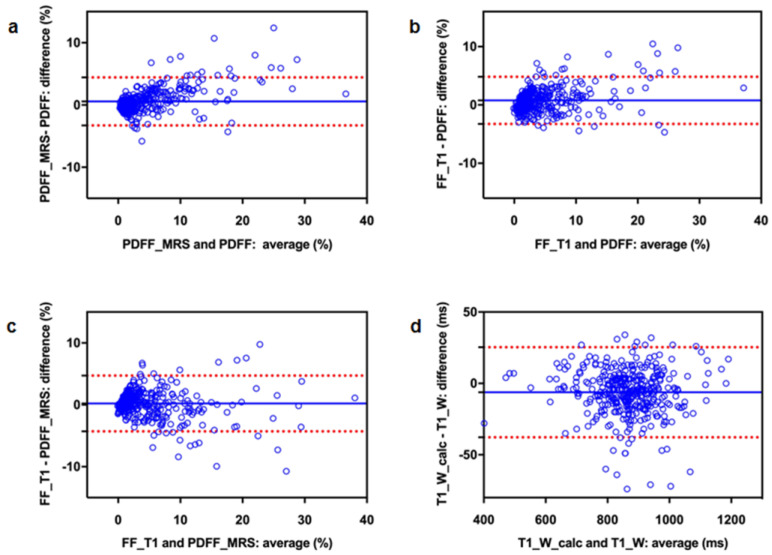
Bland–Altman plots to compare three different methods for fat quantification (**a**–**c**) as well as measured and retrospectively calculated T1_W (d): (**a**) PDFF_MRS vs. PDFF, (**b**) FF_T1 vs. PDFF, (**c**) FF_T1 vs. PDFF_MRS and (**d**) T1_W_calc vs. T1_W. The blue line represents the mean difference, the red dotted lines indicate the 95% confidence interval.

Bland–Altman analysis revealed a slightly higher fat fraction from PDFF_MRS compared to PDFF (mean bias 0.57) and from FF_T1 compared to PDFF (mean bias 0.78); FF_T1 was also somewhat higher than PDFF_MRS (mean bias 0.21) (Table 2, Figure 4). Bland–Altman plots showed a trend towards larger deviations for higher fat content (Figure 4).

T1_W measured from 2-point Dixon and T1_W_calc, which was calculated using T1_in and PDFF, demonstrated an excellent correlation (R = 0.986, *p* < 0.001). Descriptive statistics for T1_in, T1_W, and T1_W_calc are given in Table 1. Bland–Altman analysis yielded a minor underestimation of T1_W_calc relative to T1_W (mean bias: 6.23, 95% CI: −25.3, 37.8) (Figure 4).

## 4. Discussion

Using a 3D VFA technique with high parallel imaging acceleration factor, T1 mapping of the whole liver was feasible within a single breathhold with acceptable spatial resolution. Two echoes were acquired in our prototype sequence to calculate T1 maps from a water and fat in-phase signal (T1_in) and water-only signal (T1_W) based on the Dixon technique. Thus far, most authors who employed the VFA technique for T1 quantification in the liver applied only a single echo time [8,9,12,13,14,18,19,31]. For the sake of acquisition time, TE is usually set as short as possible ending up with an echo time which is near the opposed phase [8,9,17] or somewhere between the in- and opposed-phase condition [12,14].

In our study including 386 patients, we were able to demonstrate that T1_in and T1_W were significantly different and that their difference is significantly correlated with the fat fraction in the liver.

Thus far, the role of fat in T1 mapping of the liver has only been discussed in a few papers: Dixon water-only T1 mapping is described as a more reliable estimation of T1 in the presence of fat for dynamic contrast-enhanced MRI [22] and for the evaluation of liver capacity using plain as well as gadoxetic acid-enhanced T1 mapping of the liver [24].

Besides the VFA technique, inversion recovery-based sequences such as Look-Locker [2,16] or Modified Look-Locker Inversion recovery (MOLLI) [5,23] have been used for T1 quantification in the liver. Depending on TR, Mozes et al. showed prolonged T1 in the presence of increased hepatic fat [23]. Liu and coworkers reported that the influence of fat on liver T1 will be reduced at 3 T using a TE of 1.8 ms [32]. In cardiac imaging, where MOLLI is usually applied for T1 quantification of the myocardium, fat might also influence the T1 results—as intramyocardial fat or by partial volume effects at tissue boundaries: Kellman and coworkers reported an additive or subtractive T1 bias for the myocardium, depending on whether the center frequency corresponds to the myocardium and fat being in-phase or out-of-phase [33]. In skeletal muscle imaging, T1 mapping has been proposed for monitoring fatty infiltrations in neuromuscular disorders, as T1 decreased with increasing intramuscular fat fraction [34].

More recent studies emphasize the influence of fat on T1 quantification of the liver [31] and new techniques addressing the fat–water separation problem are described [35,36].

Based on those previous results and our current results, it can be concluded that the T1 bias caused by increased hepatic fat will be especially relevant when unenhanced T1 mapping is used to estimate liver function or to evaluate liver fibrosis. T1 values acquired in the hepatobiliary phase after the application of Gd-EOB-DTPA are significantly shorter than the precontrast values; thus, one might assume a minor influence of fat in that case. However, parameters combining pre- and postcontrast T1 (e.g., T1 reduction rate) which are mainly used in Gd-EOB-enhanced quantitative liver imaging [2,5,8,9,10,11,12,14,15,16,17,19,24,37] will also be affected.

In our present study, we introduce a novel parameter FF_T1 which determines the variation of T1_W relative to T1_in; its definition was motivated by the low and constant T1 of fat. Because of the definition of FF_T1, negative values can occur—although they are meaningless from a physiological point of view. Negative values for FF_T1 (up to −0.77%), which were found in our collective in eight patients, have to be interpreted as a very low fat fraction. The term “fat fraction” for the newly defined parameter FF_T1 seems to be justified—at least as an estimation of fat content—for the following reasons: FF_T1 showed a significant correlation with PDFF and both measures even yielded similar absolute values. Yokoo et al. recently published a meta-analysis which demonstrated excellent linearity, bias and precision of PDFF across field strengths, imager manufacturers, and reconstruction methods [21]. Similar to that work, we found a very good correlation between PDFF and PDFF_MRS with a minimal underestimation of PDFF. The same applies to FF_T1 and PDFF_MRS, but with a minimal overestimation of FF_T1.

With the empirically found correlation of PDFF and FF_T1, it is even possible to estimate T1_W retrospectively (T1_W_calc) from PDFF and T1_in. T1_W_calc was computed for all patients and compared to the measured T1_W from two-point Dixon T1 mapping. It is worth emphasizing that a data-focused approach was utilized here. With the VFA T1 mapping as well as the multi-echo Dixon method being based on the FLASH signal model, the effective T1 of the in-phase echo, i.e., T1_in, can be calculated from T1_W, T1_F and PDFF. Furthermore, this dependency also includes the protocol parameters of the VFA acquisitions and even potentially details of the fitting method. With the assumption of T1_F to be known, it is even conceivable that T1_W can be determined from PDFF and an effective T1 determined at a given echo time. A more detailed analysis is beyond the scope of this manuscript but may warrant future investigations.

Considering FF_T1 as a measure of the fat fraction, retrospective calculation of T1_W is possible using T1_in and PDFF. T1_W measured from our two-point Dixon technique and T1_W_calc yielded excellent correlation with only a minor bias. Therefore, T1_W can be estimated retrospectively if the fat fraction is known from a separate acquisition.

Several limitations of our sequence technique and our study design in this single-center study have to be addressed. Global fat–water swaps were a limitation of our VFA technique combined with a two-point Dixon method, as T1_W maps were confounded in about 4% of patients. The number of swaps was smaller than reported in an earlier study of a multi-echo Dixon technique for hepatic fat and iron quantification [38]. As R2* correction and spectral fat modeling are not included, FF_T1 will not be able to quantify hepatic fat exactly. Although FF_T1 showed a very good correlation with PDFF and PDFF_MRS in our study collective, there was a trend towards larger errors for higher fat fractions and the number of patients with high hepatic fat was limited in our collective: using the grading results of França et al., 23 patients with severe steatosis (PDFF > 12.9%) [39] were included in our study. According to Tang and coworkers, there were only four patients with severe steatosis (PDFF > 22.2%) [40]. Furthermore, patients with R2* > 200 s^−1^ have been explicitly excluded from our study. A follow-up of our study focusing on patients with severe hepatic iron overload and/or severe steatosis might be very interesting but needs adaption/optimization of our techniques for T1, PDFF and R2* mapping.

Another limitation of our study was the way the data were collected and evaluated. In contrast to PDFF and FF_T1, which were evaluated in a single two-dimensional circular ROI, PDFF_MRS was measured in a co-localized voxel. Furthermore, all three measurements were acquired in separate breathholds. These facts will reduce the accuracy of individual results and might partially explain some spread of the results.

## 5. Conclusions

T1 calculated from the in-phase signal (T1_in) in a VFA technique is significantly different from T1 calculated from the water-only signal (T1_W) and the difference is significantly correlated with the fat fraction. Therefore, T1 based on a VFA method will yield different results depending on TE and this effect might be especially important for T1 quantification in patients with steatosis. Furthermore, it has been shown that T1_W can be estimated retrospectively from T1_in and PDFF in those cases where T1_W cannot be (or was not) assessed using a Dixon technique.

## Figures and Tables

**Table 1:** Descriptive statistics for T1 relaxation times (T1_in, T1_W, T1_W-T1_in, T1_W_calc), liver fat measurements (PDFF, PDFF_MRS, FF_T1) and R2* in 386 patients.

	Mean ± Standard Deviation	Range (Min, Max)
T1_in (ms)	835 ± 102	448, 1172
T1_W (ms)	873 ± 96	611, 1183
T1_W-T1_in (ms)	38 ± 38	−7, 225
T1_W_calc (ms)	867 ± 95	608, 1199
PDFF (%)	4.11 ± 4.73	0.1, 35.7
PDFF_MRS (%)	4.68 ± 5.87	0.0, 37.5
FF_T1 (%)	4.88 ± 5.46	−0.77, 38.6
R2* (s^−1^)	52.0 ± 21.5	2.0, 198.3

T1_in: T1 relaxation time from fat and water in-phase signal; T1_W: T1 relaxation time from water-only signal; T1_W_calc: T1 relaxation time from water-only signal, calculated using T1_in and PDFF; PDFF: proton density fat fraction (from MR imaging); PDFF_MRS: proton density fat fraction (from MR spectroscopy); FF_T1: fat fraction, calculated from T1 mapping combined with a 2-point Dixon method; R2*: relaxation rate.

**Table 2:** Pearson correlation coefficient R and Bland–Altman analysis for liver fat measurements (PDFF, PDFF_MRS, FF_T1).

	R	Mean Bias	95% CI
PDFF_MRS vs. PDFF FF_T1 vs. PDFF FF_T1 vs. PDFF_MRS	0.954	0.57	−3.29, 4.43
	0.930	0.78	−3.27, 4.82
	0.922	0.21	−4.30, 4.71

PDFF: proton density fat fraction (from MR imaging); PDFF_MRS: proton density fat fraction (from MR spectroscopy); FF_T1: fat fraction, calculated from T1 mapping combined with a 2-point Dixon method; CI: confidence interval.

## Data Availability

The source data presented in this study are available on request from the corresponding author. The source data are not publicly available due to patients’ privacy.

## References

[1] Cheng H.L., Stikov N., Ghugre N.R., Wright G.A. (2012). Practical medical applications of quantitative MR relaxometry. J. Magn. Reson. Imaging.

[2] Katsube T., Okada M., Kumano S., Hori M., Imaoka I., Ishii K., Kudo M., Kitagaki H., Murakami T. (2011). Estimation of Liver Function Using T1 Mapping on Gd-EOB-DTPA-Enhanced Magnetic Resonance Imaging. Invest. Radiol..

[3] Heye T., Yang S.-R., Bock M., Brost S., Weigand K., Longerich T., Kauczor H.-U., Hosch W. (2012). MR relaxometry of the liver: Significant elevation of T1 relaxation time in patients with liver cirrhosis. Eur. Radiol..

[4] Cassinotto C., Feldis M., Vergniol J., Mouries A., Cochet H., Lapuyade B., Hocquelet A., Juanola E., Foucher J., Laurent F. (2015). MR relaxometry in chronic liver diseases: Comparison of T1 mapping, T2 mapping, and diffusion-weighted imaging for assessing cirrhosis diagnosis and severity. Eur. J. Radiol..

[5] Yoon J.H., Lee J.M., Paek M., Han J.K., Choi B.I. (2016). Quantitative assessment of hepatic function: Modified look-locker inversion recovery (MOLLI) sequence for T1 mapping on Gd-EOB-DTPA-enhanced liver MR imaging. Eur. Radiol..

[6] Fahlenkamp U.L., Kunkel J., Ziegeler K., Neumann K., Adams L.C., Engel G., Böker S., Makowski M.R. (2021). Correlation of Native Liver Parenchyma T1 and T2 Relaxation Times and Liver Synthetic Function Tests: A Pilot Study. Diagnostics.

[7] Hamm B., Staks T., Mühler A., Bollow M., Taupitz M., Frenzel T., Wolf K.J., Weinmann H.J., Lange L. (1995). Phase I clinical evaluation of Gd-EOB-DTPA as a hepatobiliary MR contrast agent: Safety, pharmacokinetics, and MR imaging. Radiology.

[8] Kamimura K., Fukukura Y., Yoneyama T., Takumi K., Tateyama A., Umanodan A., Shindo T., Kumagae Y., Ueno S.-I., Koriyama C. (2014). Quantitative evaluation of liver function with T1 relaxation time index on Gd-EOB-DTPA-enhanced MRI: Comparison with signal intensity-based indices. J. Magn. Reson. Imaging.

[9] Yoneyama T., Fukukura Y., Kamimura K., Takumi K., Umanodan A., Ueno S., Nakajo M. (2014). Efficacy of liver parenchymal enhancement and liver volume to standard liver volume ratio on Gd-EOB-DTPA-enhanced MRI for estimation of liver function. Eur. Radiol..

[10] Haimerl M., Schlabeck M., Verloh N., Zeman F., Fellner C., Nickel D., Barreiros A.P., Loss M., Stroszczynski C., Wiggermann P. (2016). Volume-assisted estimation of liver function based on Gd-EOB-DTPA–enhanced MR relaxometry. Eur. Radiol..

[11] Haimerl M., Verloh N., Zeman F., Fellner C., Nickel D., Lang S.A., Teufel A., Stroszczynski C., Wiggermann P. (2017). Gd-EOB-DTPA-enhanced MRI for evaluation of liver function: Comparison between signal-intensity-based indices and T1 relaxometry. Sci. Rep..

[12] Besa C., Bane O., Jajamovich G., Marchione J., Taouli B. (2015). 3D T1 relaxometry pre and post gadoxetic acid injection for the assessment of liver cirrhosis and liver function. Magn. Reson. Imaging.

[13] Yoon J.H., Lee J.M., Kim E., Okuaki T., Han J.K. (2017). Quantitative Liver Function Analysis: Volumetric T1 Mapping with Fast Multisection B_1_ Inhomogeneity Correction in Hepatocyte-specific Contrast-enhanced Liver MR Imaging. Radiology.

[14] Ding Y., Rao S.-X., Chen C., Li R., Zeng M.S. (2015). Assessing liver function in patients with HBV-related HCC: A comparison of T1 mapping on Gd-EOB-DTPA-enhanced MR imaging with DWI. Eur. Radiol..

[15] Haimerl M., Utpatel K., Verloh N., Zeman F., Fellner C., Nickel D., Teufel A., Fichtner-Feigl S., Evert M., Stroszczynski C. (2017). Gd-EOB-DTPA-enhanced MR relaxometry for the detection and staging of liver fibrosis. Sci. Rep..

[16] Pan S., Wang X.Q., Guo Q.Y. (2018). Quantitative assessment of hepatic fibrosis in chronic hepatitis B and C: T1 mapping on Gd-EOB-DTPA-enhanced liver magnetic resonance imaging. World J. Gastroenterol..

[17] Peng Z., Li C., Chan T., Cai H., Luo Y., Dong Z., Li Z.-P., Feng S.-T. (2017). Quantitative evaluation of Gd-EOB-DTPA uptake in focal liver lesions by using T1 mapping: Differences between hepatocellular carcinoma, hepatic focal nodular hyperplasia and cavernous hemangioma. Oncotarget.

[18] Wang F., Yang Q., Zhang Y., Liu J., Liu M., Zhu J. (2022). 3D variable flip angle T1 mapping for differentiating benign and malignant liver lesions at 3T: Comparison with diffusion weighted imaging. BMC Med. Imaging.

[19] Wang W.-T., Zhu S., Ding Y., Yang L., Chen C.-Z., Ye Q.-H., Ji Y., Zeng M.-S., Rao S.-X. (2018). T1 mapping on gadoxetic acid-enhanced MR imaging predicts recurrence of hepatocellular carcinoma after hepatectomy. Eur. J. Radiol..

[20] Haimerl M., Utpatel K., Götz A., Zeman F., Fellner C., Nickel D., Luerken L., Brennfleck F., Stroszczynski C., Scheiter A. (2021). Quantification of contrast uptake in the hepatobiliary phase helps to differentiate hepatocellular carcinoma grade. Sci. Rep..

[21] Yokoo T., Serai S.D., Pirasteh A., Bashir M.R., Hamilton G., Hernando D., Hu H.H., Hetterich H., Kühn J.-P., Kukuk G.M. (2018). Linearity, Bias, and Precision of Hepatic Proton Density Fat Fraction Measurements by Using MR Imaging: A Meta-Analysis. Radiology.

[22] Le Y., Dale B., Akisik F., Koons K., Lin C. (2016). Improved T1, contrast concentration, and pharmacokinetic parameter quantification in the presence of fat with two-point dixon for dynamic contrast-enhanced magnetic resonance imaging. Magn. Reson. Med..

[23] Mozes F.E., Tunnicliffe E.M., Pavlides M., Robson M.D. (2016). Influence of fat on liver *T*_1_ measurements using modified Look-Locker inversion recovery (MOLLI) methods at 3T. J. Magn. Reson. Imaging.

[24] Haimerl M., Probst U., Poelsterl S., Fellner C., Nickel D., Weigand K., Brunner S.M., Zeman F., Stroszczynski C., Wiggermann P. (2018). Evaluation of two-point Dixon water-fat separation for liver specific contrast-enhanced assessment of liver maximum capacity. Sci. Rep..

[25] Fram E.K., Herfkens R.J., Johnson G.A., Glover G.H., Karis J.P., Shimakawa A., Perkins T.G., Pelc N.J. (1987). Rapid calculation of T1 using variable flip angle gradient refocused imaging. Magn. Reson. Imaging.

[26] Dixon W.T. (1984). Simple proton spectroscopic imaging. Radiology.

[27] Breuer F.A., Blaimer M., Mueller M.F., Seiberlich N., Heidemann R.M., Griswold M.A., Jakob P.M. (2006). Controlled aliasing in volumetric parallel imaging (2D CAIPIRINHA). Magn. Reson. Med..

[28] Zhong X., Nickel M.D., Kannengiesser S.A., Dale B.M., Kiefer B., Bashir M.R. (2014). Liver fat quantification using a multi-step adaptive fitting approach with multi-echo GRE imaging. Magn. Reson. Med..

[29] Pineda N., Sharma P., Xu Q., Hu X., Vos M., Martin D.R. (2009). Measurement of Hepatic Lipid: High-Speed T2-Corrected Multiecho Acquisition at ^1^H MR Spectroscopy—A Rapid and Accurate Technique. Radiology.

[30] Giavarina D. (2015). Understanding Bland Altman analysis. Biochem. Med..

[31] Ahn J.-H., Yu J.-S., Park K.-S., Kang S.H., Huh J.H., Chang J.S., Lee J.-H., Kim M.Y., Nickel M.D., Kannengiesser S. (2021). Effect of hepatic steatosis on native T1 mapping of 3T magnetic resonance imaging in the assessment of T1 values for patients with non-alcoholic fatty liver disease. Magn. Reson. Imaging.

[32] Liu C.-Y., Noda C., Ambale-Venkatesh B., Kassai Y., Bluemke D., Lima J.A.C. (2022). Evaluation of liver T1 using MOLLI gradient echo readout under the influence of fat. Magn. Reson. Imaging.

[33] Kellman P., Bandettini W.P., Mancini C., Hammer-Hansen S., Hansen M.S., Arai A.E. (2015). Characterization of myocardial T1-mapping bias caused by intramyocardial fat in inversion recovery and saturation recovery techniques. J. Cardiovasc. Magn. Reson..

[34] Marty B., Coppa B., Carlier P.G. (2018). Monitoring skeletal muscle chronic fatty degenerations with fast T1-mapping. Eur. Radiol..

[35] Peng H., Cheng C., Wan Q., Jia S., Wang S., Lv J. (2022). Fast multi-parametric imaging in abdomen by B1+ corrected dual-flip angle sequence with interleaved echo acquisition. Magn. Reson. Med..

[36] Feng L., Liu F., Soultanidis G., Liu C., Benkert T., Block K.T., Fayad Z.A., Yang Y. (2021). Magnetization-prepared GRASP MRI for rapid 3D T1 mapping and fat/water-separated T1 mapping. Magn. Reson. Med..

[37] Haimerl M., Fuhrmann I., Poelsterl S., Fellner C., Nickel M.D., Weigand K., Dahlke M.H., Verloh N., Stroszczynski C., Wiggermann P. (2018). Gd-EOB-DTPA-enhanced T1 relaxometry for assessment of liver function determined by real-time 13C-methacetin breath test. Eur. Radiol..

[38] Henninger B., Zoller H., Kannengiesser S., Zhong X., Jaschke W., Kremser C. (2017). 3D Multiecho Dixon for the Evaluation of Hepatic Iron and Fat in a Clinical Setting. J. Magn. Reson. Imaging.

[39] França M., Alberich-Bayarri Á., Martí-Bonmatí L., Oliveira P., Costa F.E., Porto G., Vizcaíno J.R., Gonzalez J.S., Ribeiro E., Oliveira J. (2017). Accurate simultaneous quantification of liver steatosis and iron overload in diffuse liver diseases with MRI. Abdom. Radiol. NY Imaging.

[40] Tang A., Tanya W., Sun M., Hamilton G., Bydder M., Wolfson T., Gamst A.C., Middleton M., Brunt E.M., Loomba R. (2013). Nonalcoholic Fatty Liver Disease: MR Imaging of Liver Proton Density Fat Fraction to Assess Hepatic Steatosis. Radiology.

